# Association between IoT-driven dynamic pulmonary function monitoring and quality of life in community-dwelling patients with chronic obstructive pulmonary disease: a 2-year longitudinal study

**DOI:** 10.3389/fmed.2026.1856422

**Published:** 2026-06-10

**Authors:** Mingming Zhao, Haixia Zheng, Liangfeng Yang, Minmin Yang, Yu Lin, Miaomiao Kong, Nan Wang, Yanping Hang, Yuguo Zhao

**Affiliations:** 1 Department of Pulmonary and Critical Care Medicine, Nanjing Gaochun People’s Hospital, Nanjing, China; 2 China Hospital Reform and Development Research Institute, Nanjing University, Nanjing, China; 3 Department of Respiratory Medicine, Nanjing Lishui District People’s Hospital, Nanjing, China

**Keywords:** chronic obstructive pulmonary disease, dynamic pulmonary function monitoring, internet of things, predictive value, quality of life

## Abstract

**Objective:**

To characterize dynamic changes in Internet of Things (IoT)-driven pulmonary function and examine their impact and predictive value on quality of life (QoL) in patients with chronic obstructive pulmonary disease (COPD).

**Methods:**

Using multistage stratified cluster random sampling, 3,000 residents with COPD high-risk factors were screened between January 2023 and January 2024 in this single-arm observational cohort study, and 360 patients who fulfilled the contemporaneous GOLD diagnostic criteria for COPD and had complete longitudinal monitoring data were included in the final analysis. All participants underwent 2-year IoT-based dynamic monitoring of forced vital capacity (FVC), forced expiratory volume in 1 s (FEV_1_), FEV_1_/FVC, peak expiratory flow (PEF), and maximal voluntary ventilation (MVV), and rates of change were calculated. Quality of life (QoL) was assessed using the WHOQOL-BREF questionnaire, and participants were stratified into good QoL (*n* = 184) and poor QoL (*n* = 176) groups according to the total score. Univariate analysis, multivariable logistic regression, and receiver operating characteristic (ROC) curve analyses were performed.

**Results:**

After 2 years, physical/environmental scores increased, while psychological/social relationship and total QoL scores decreased (*P* < 0.001). FVC, FEV_1_, FEV_1_/FVC, PEF, and MVV improved from baseline (*P* < 0.001). Smoking status, cumulative smoking load, occupational dust/harmful gas exposure, and biomass fuel use were independently associated with poorer QoL, whereas rates of change in FVC, FEV_1_, and MVV were independently associated with better QoL (*P* < 0.05). The combined model (AUC = 0.834) outperformed behavioral factor model and dynamic physiological indicator model (*P* < 0.05).

**Conclusion:**

Dynamic pulmonary function changes obtained via IoT, particularly rates of change in FVC, FEV_1_, and MVV, are independently associated with QoL in COPD. Combining these indicators with behavioral and exposure factors was associated with improved predictive accuracy for poor QoL, supporting risk stratification and individualized management.

## 1 Introduction

Chronic obstructive pulmonary disease (COPD) is a prevalent and heterogeneous chronic respiratory disorder characterized by persistent and often progressive airflow limitation, chronic respiratory symptoms, and substantial exposure-related pulmonary abnormalities. It is distinguished by its high prevalence, protracted disease course, frequent acute exacerbations, complex comorbidities, and substantial disease burden (1–3). Beyond hard outcomes such as mortality, hospitalization rates, and acute exacerbation frequency, patient quality of life (QoL) has emerged as a pivotal outcome measure for evaluating the effectiveness of comprehensive disease management throughout the illness trajectory (4, 5). Under traditional follow-up models, clinicians primarily collect intermittent, low-frequency data during clinic visits, which often fails to capture patients’ real-world respiratory physiological fluctuations, symptom burden, and behavioral adherence in daily life. This limitation frequently results in delayed risk assessment and intervention timing. Recently, the rapid advancement of Internet of Things (IoT) in chronic disease management, featuring wearable and portable sensors as the front-end, mobile internet as the communication channel, cloud/edge computing providing computational power, and physician decision support closing the loop, offers novel possibilities for continuous monitoring, stratified intervention, and remote management of COPD (6–8).

Unlike the snapshot measurements provided by traditional pulmonary function tests, IoT enables the continuous capture and transmission of multimodal respiratory data under real-world, long-term, low-burden conditions. This includes parameters such as forced vital capacity (FVC), forced expiratory volume in 1 s (FEV_1_), FEV_1_/FVC ratio, peak expiratory flow (PEF), and maximal voluntary ventilation (MVV). Furthermore, this capability can be augmented with data on oxygen saturation, pulse rate, physical activity levels, sleep patterns, environmental exposures (e.g., temperature, humidity, PM_2_._5_), and patient-reported symptoms (9). At the data level, these high-frequency, longitudinal time series facilitate the delineation of individualized lung function trajectories and circadian rhythms, aiding in the identification of transitional phases from stability to fluctuation to decompensation. At the algorithmic level, techniques for detecting abnormal trends (e.g., sliding window analysis, threshold-based alerts, change point detection) and comparisons against individual baselines can proactively trigger yellow-light warnings. At the service level, IoT platforms empower healthcare providers to remotely deliver health education, medication guidance, inhaler technique correction, and follow-up supervision. They also enable graded referral and rapid consultation for high-risk individuals, thereby extending hospital-centric management toward person/family-centered continuous care (10). Notably, compared with single-point static pulmonary function measurements, dynamic changes captured through continuous monitoring may better reflect disease control trends, changes in functional reserve, and long-term outcome risks, offering a more sensitive quantitative basis for assessing patient QoL.

QoL represents a crucial endpoint for assessing the value of IoT interventions. Scales such as the World Health Organization Quality of Life questionnaire-abbreviated version (WHOQOL-BREF) comprehensively evaluate patients’ subjective health experiences across four domains: physical, psychological, social relationships, and environment (11–13). However, QoL is influenced not only by disease severity but also by the complex interplay of factors including lifestyle habits (smoking and secondhand smoke exposure), occupational and household environments (dust/harmful gases, biomass fuel use, ventilation), social support, and psychological resilience. The unique value of IoT lies in three key aspects: First, it enables the temporal alignment of objective physiological parameters, subjective symptoms, and behavioral/environmental exposures, thereby improving the precision of individualized risk identification. Second, it facilitates precision management through a monitoring-feedback-intervention-reassessment closed loop, significantly shortening the decision-making lag from risk exposure to clinical action. Third, it improves adherence and self-management capabilities via patient apps providing immediate feedback, goal setting, and behavioral incentives, which theoretically contributes to improved lung function stability and patient QoL (14).

Although existing studies progressively suggest that remote monitoring and digital interventions may reduce acute exacerbations and emergency department visits, substantial heterogeneity in benefits persists across different populations and endpoints, particularly QoL. Contributing factors may include variations in devices and algorithms, sampling frequency and data completeness, patient digital literacy and engagement levels, intervention intensity, and multidisciplinary collaboration. Furthermore, previous research has largely focused on outcomes such as acute exacerbations, hospital readmissions, or healthcare resource utilization, whereas longitudinal evidence regarding the associations between dynamic lung function changes and QoL outcomes remains relatively limited. Therefore, it is necessary to explore, through systematic data collection and rigorous statistical modeling, whether dynamic physiological indicators obtained via IoT-based monitoring are associated with QoL outcomes in COPD patients, and to further clarify the potential roles of modifiable factors such as smoking burden and occupational or household exposures. To address this gap, the present study was conducted in a community-based COPD cohort using an IoT platform for long-term, continuous, multi-indicator pulmonary function monitoring and follow-up. A standardized QoL instrument was applied for multidimensional assessment, and multivariable modeling together with receiver operating characteristic (ROC) curve analysis was performed to evaluate the associations and predictive value of smoking-related factors, occupational or household exposures, and dynamic physiological indicators for QoL outcomes. The present study was designed as a hypothesis-generating observational cohort study intended to provide observational evidence regarding the relationships between IoT-driven dynamic pulmonary function trajectories and QoL outcomes in COPD patients, with the aim of supporting future risk stratification and individualized management strategies in primary care settings.

## 2 Materials and methods

### 2.1 Study subjects

Using a multistage stratified cluster random sampling method, approximately 3,000 residents with high-risk factors for COPD were recruited from township central health centers across Gaochun District between January 2023 and January 2024 as the study population.

### 2.2 Inclusion and exclusion criteria

The inclusion criteria were as follows: (1) mentally competent individuals capable of cooperating with pulmonary function testing; (2) presence of at least one of the following COPD high-risk factors: a history of smoking (regardless of current smoking status), symptoms such as chronic cough, phlegm when coughing or wheezing, occupational exposure such as dust exposure, a family history of COPD or asthma, or a COPD Screening Questionnaire (COPD-SQ) score ≥ 16; (3) fulfillment of the contemporaneous GOLD diagnostic criteria available during the study period, including post-bronchodilator FEV_1_/FVC < 0.70 after exclusion of other known chronic pulmonary diseases; and (4) provision of written informed consent. Exclusion criteria comprised: (1) comorbid major cardiocerebrovascular diseases or psychiatric disorders rendering the individual unable to undergo testing; (2) recent history of thoracic or abdominal surgery; (3) history of acute lower respiratory tract infection, cardiac arrhythmia, or related conditions within the preceding month; (4) previously established diagnoses of other chronic pulmonary diseases, including bronchial asthma, bronchiectasis, or interstitial lung disease.

### 2.3 Diagnostic criteria for COPD

The diagnosis of COPD was established in accordance with the contemporaneous Global Initiative for GOLD criteria available during the study period (15), using post-bronchodilator FEV_1_/FVC < 0.70 in combination with respiratory symptoms and exposure-related risk factors, including smoking history and occupational exposure. Participants demonstrating a post-bronchodilator FEV_1_/FVC ratio of < 70% were identified as having persistent airflow limitation. Following the exclusion of other known pulmonary diseases, this finding confirmed the diagnosis of COPD.

### 2.4 Research methods

#### 2.4.1 Survey investigation

General data for all participants were collected using a standardized hospital patient information questionnaire. This encompassed gender, age, educational attainment, marital status, occupation, place of residence, smoking status, secondhand smoke exposure, history of occupational dust exposure, exposure to harmful gases, installation of kitchen ventilation equipment, living conditions, and family history of respiratory diseases. Smoking was defined as current or former habitual use of any tobacco product (16). Secondhand smoke exposure was defined as non-smokers being exposed to smoke exhaled by smokers for ≥ 1 day per week (17). Pulmonary function testing was performed using standardized portable spirometric devices (X1 portable pulmonary function detector, XEEK Medical Instrument Co., Ltd., Xiamen, China) by dedicated personnel who had received certified and standardized training in pulmonary function assessment. All spirometric procedures were conducted in accordance with the American Thoracic Society/European Respiratory Society (ATS/ERS) recommendations to ensure acceptable reproducibility and quality control standards. Participants first underwent baseline spirometric testing, followed by a bronchodilator reversibility test involving inhaled bronchodilator administration. Ventilatory parameters, including FEV_1_ and FVC, were collected both before bronchodilator administration and 15 min after inhalation. Repeated maneuvers were performed when necessary to ensure measurement reliability and acceptable spirometric quality grades.

Based on the pulmonary function test results and the predefined diagnostic criteria for GOLD, 360 participants were ultimately diagnosed with COPD. All 360 COPD patients received IoT-enabled COPD support, implemented as follows: Project team members visited participants’ homes to assist in configuring wearable devices designated for COPD screening. Participants then inputted basic information via a smartphone app. They were instructed to wear the physiological parameter monitors at home. Health data captured by these monitors were automatically transmitted to the participants’ app and could be relayed via Bluetooth technology to the physicians’ app terminal. The physician-facing app terminal provided integrated health services, including remote health consultations, health information collection, alerts and emergency response management for abnormal findings, tiered referral coordination, and specialist consultation. Leveraging the real-time monitoring data displayed on the platform, physicians confirmed the COPD diagnosis and delivered personalized, evidence-based health education or medication guidance to each patient. Key spirometric indices (FVC, FEV_1_, FEV_1_/FVC ratio, PEF, and MVV) were recorded at baseline (enrollment) and study conclusion. To evaluate the impact of longitudinal changes in IoT-based dynamic monitoring indicators on prognosis, the rate of change for each indicator (FVC, FEV_1_, FEV_1_/FVC ratio, PEF, and MVV) over the 2-year follow-up period was calculated using the formula: [(final value – baseline value)/baseline value × 100%].

**FIGURE 1:**
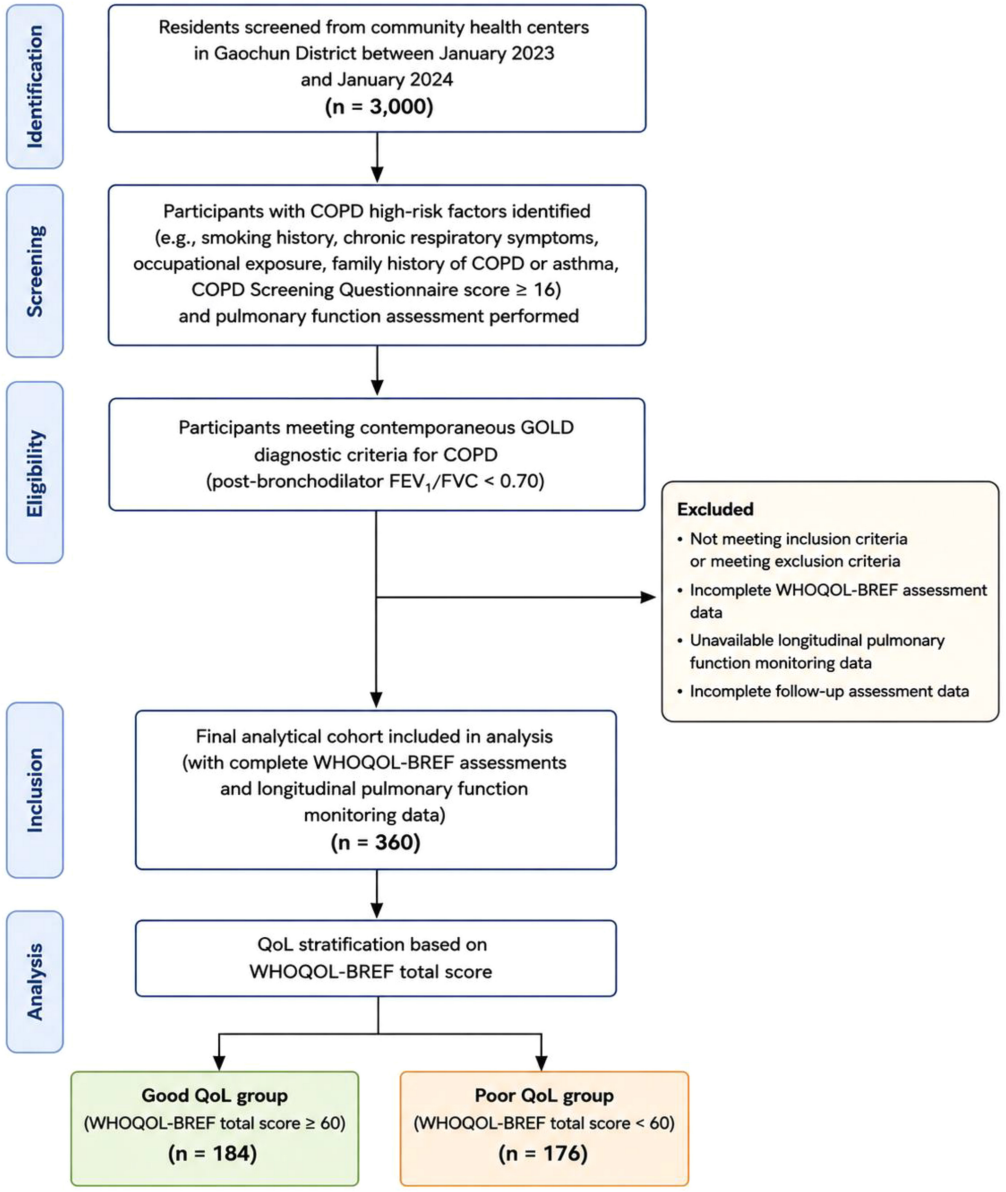
STROBE-compliant participant flow diagram.

Confirmed COPD patients were followed for 2 years. As this was a single-arm observational cohort study without a concurrent control group, the findings should be interpreted as associative rather than causal. QoL was assessed at baseline and the 2-year endpoint using the WHOQOL-BREF (18). This instrument comprises 24 items distributed across four domains: physical health (7 items, score range 7–35 points), psychological health (6 items, score range 6–30 points), social relationships (3 items, score range 3–15 points), and environment (8 items, score range 8–40 points). Items were rated on a 5-point Likert scale, yielding a total score range of 24– 120 points, with higher scores indicating better QoL. Based on the total WHOQOL-BREF score at the 2-year follow-up, patients were categorized into two groups: a good QoL group (total score exceeding the mean score) and a poor QoL group (total score equal to or below the mean score). Given the absence of a universally validated WHOQOL-BREF cut-off value for COPD populations in community-based longitudinal monitoring settings, the sample mean score was used to facilitate exploratory risk stratification and multivariable model construction. A STROBE-compliant participant flow diagram has been added to illustrate patient screening, enrollment, follow-up, and final inclusion in the analysis (Figure 1).

### 2.5 Statistical analysis

Data analysis was performed using SPSS Statistics software, version 24.0 (IBM Corp., Armonk, NY, United States). Continuous variables conforming to a normal distribution were presented as mean ± standard deviation (SD). Comparisons between the two QoL groups utilized the independent samples *t*-test, while within-group comparisons (pre- vs. post-intervention) employed the paired *t*-test. Continuous variables not adhering to a normal distribution were expressed as median and interquartile range (IQR), reported as M (P_25_, P_75_), and analyzed using the rank-sum test for between-group comparisons. Categorical data were presented as counts (n), with group differences assessed using the Chi-square (χ^2^) test. Univariate analyses were performed as exploratory screening procedures to identify candidate variables for subsequent multivariable modeling. No formal correction for multiple comparisons was applied at this stage; therefore, variables identified in univariate analyses were interpreted cautiously and further evaluated in multivariable regression models. Multivariable logistic regression analysis was performed to identify factors independently associated with poor QoL in COPD patients. Before multivariable logistic regression analysis, multicollinearity among independent variables was assessed using variance inflation factor (VIF) values. Variables with VIF < 5 were considered to have no significant multicollinearity. Model fitness and calibration were evaluated using the Hosmer-Lemeshow goodness-of-fit test. As a sensitivity analysis, WHOQOL-BREF total score was additionally analyzed as a continuous outcome variable using exploratory linear regression. The predictive value of these significant factors for poor QoL was further evaluated using ROC curve analysis. A *P*-value < 0.05 was considered statistically significant for all analyses. Participants with incomplete WHOQOL-BREF assessments or unavailable key pulmonary function monitoring data during follow-up were not included in the final analytical cohort. Therefore, the present study adopted a complete-case analysis framework based on participants with available longitudinal monitoring and QoL assessment data.

## 3 Results

### 3.1 COPD incidence and patient characteristics

Among the 360 patients diagnosed with COPD, males constituted 73.89% (*n* = 266) and females 26.11% (*n* = 94). The median age was 64.00 years (IQR: 58.00–70.00 years). A history of smoking was reported in 64.72% (*n* = 233) of patients. Among smokers, 66.11% (*n* = 238) had a cumulative smoking exposure of 0–30 pack-years, while 33.89% (*n* = 122) had > 30 pack-years. Secondhand smoke exposure was documented in 17.78% (*n* = 64), occupational dust/harmful gas exposure in 38.33% (*n* = 138), and biomass fuel use in 43.89% (*n* = 158). A family history of respiratory diseases was present in 32.78% (*n* = 118), and 30.56% (*n* = 110) reported a personal history of non-COPD respiratory conditions. These findings indicate that the enrolled COPD cohort predominantly comprised middle-aged and elderly males, with smoking, occupational exposures (dust/harmful gases), and biomass fuel use identified as significant associated factors within this population (Table 1).

**TABLE 1:** Clinical characteristics of the 360 COPD patients [n (%), M (P_25_, P_75_)].

Characteristic	Result
Gender	
Male	266 (73.89)
Female	94 (26.11)
Age	64.00 (58.00, 70.00)
Smoking history	233 (64.72)
Cumulative smoking	
0∼30 Pack-years	238 (66.11)
> 30 Pack-years	122 (33.89)
Secondhand smoke exposure	64 (17.78)
Occupational dust/harmful gas exposure	138 (38.33)
Biomass fuel use	158 (43.89)
Family history of respiratory diseases	118 (32.78)
Personal history of respiratory diseases	110 (30.56)

COPD, Chronic obstructive pulmonary disease.

### 3.2 Longitudinal QoL assessment in COPD patients over 2 years

**FIGURE 2:**
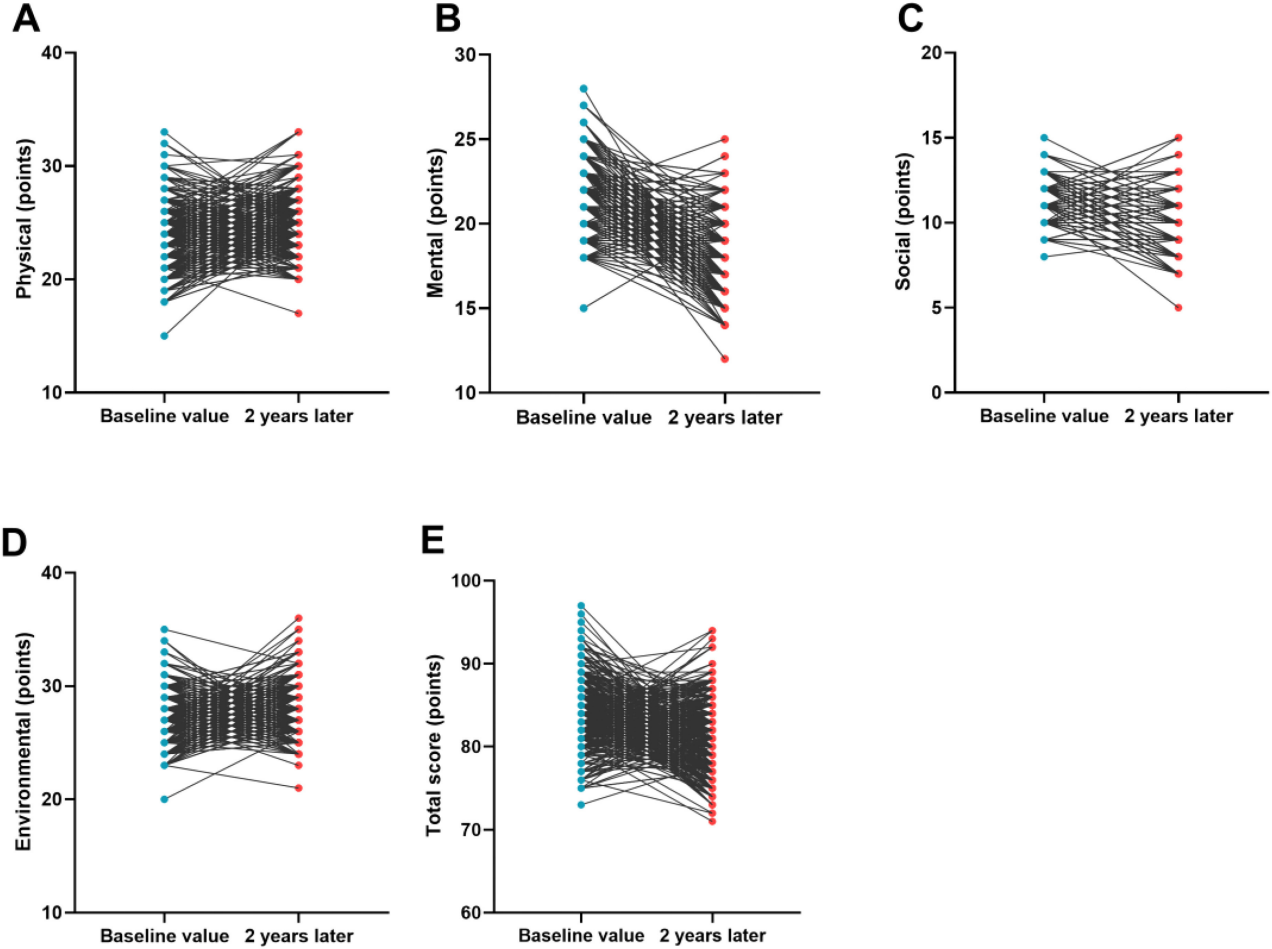
Changes in QoL between baseline and 2-year follow-up in COPD patients. **(A)** Physical health; **(B)** psychological health; **(C)** social relationships; **(D)** environment; **(E)** total QoL. QoL, Quality of life; COPD, Chronic obstructive pulmonary disease.

Results from the 2-year QoL assessment in the 360 COPD patients revealed the following median scores (IQR) at baseline: physical health domain: 24.00 (22.00, 26.00); psychological health domain: 22.00 (20.00, 23.00); social relationships domain: 11.00 (11.00, 12.00); environment domain: 27.00 (26.00, 29.00); and total QoL score: 84.00 (81.00, 86.00). At the 2-year follow-up, the corresponding scores were: physical health: 25.00 (23.00, 27.00); psychological health: 18.00 (17.00, 20.00); social relationships: 10.00 (9.00, 11.00); environment: 28.00 (26.50, 30.00); and total QoL: 82.00 (78.00, 84.50). Statistical analysis demonstrated statistically significant differences between baseline and 2-year scores for all domains and the total score (*Z* = −6.690 for physical health; *Z* = −14.424 for psychological health; *Z* = −11.487 for social relationships; *Z* = −5.091 for environment; *Z* = −6.768 for total QoL; all *P* < 0.001). Notably, scores in the physical health and environment domains showed significant improvement over the 2-year period, whereas scores in the psychological health and social relationships domains, along with the total QoL score, exhibited statistically significant deterioration (Figure 2 and Table 2).

**TABLE 2:** QoL scores in COPD patients at baseline and 2-year follow-up [M (P_25_, P_75_)].

Timepoint	Physical health	Psychological health	Social relationships	Environment	Total QoL
Baseline	24.00 (22.00, 26.00)	22.00 (20.00, 23.00)	11.00 (11.00, 12.00)	27.00 (26.00, 29.00)	84.00 (81.00, 86.00)
2-Year	25.00 (23.00, 27.00)	18.00 (17.00, 20.00)	10.00 (9.00, 11.00)	28.00 (26.50, 30.00)	82.00 (78.00, 84.50)
Z	−6.690	−14.424	−11.487	−5.091	−6.768
*P*	<0.001	< 0.001	<0.001	< 0.001	<0.001

QoL, Quality of life; COPD, Chronic obstructive pulmonary disease.

### 3.3 Longitudinal changes in IoT-driven dynamic physiological indicators in COPD patients over 2 years

**FIGURE 3:**
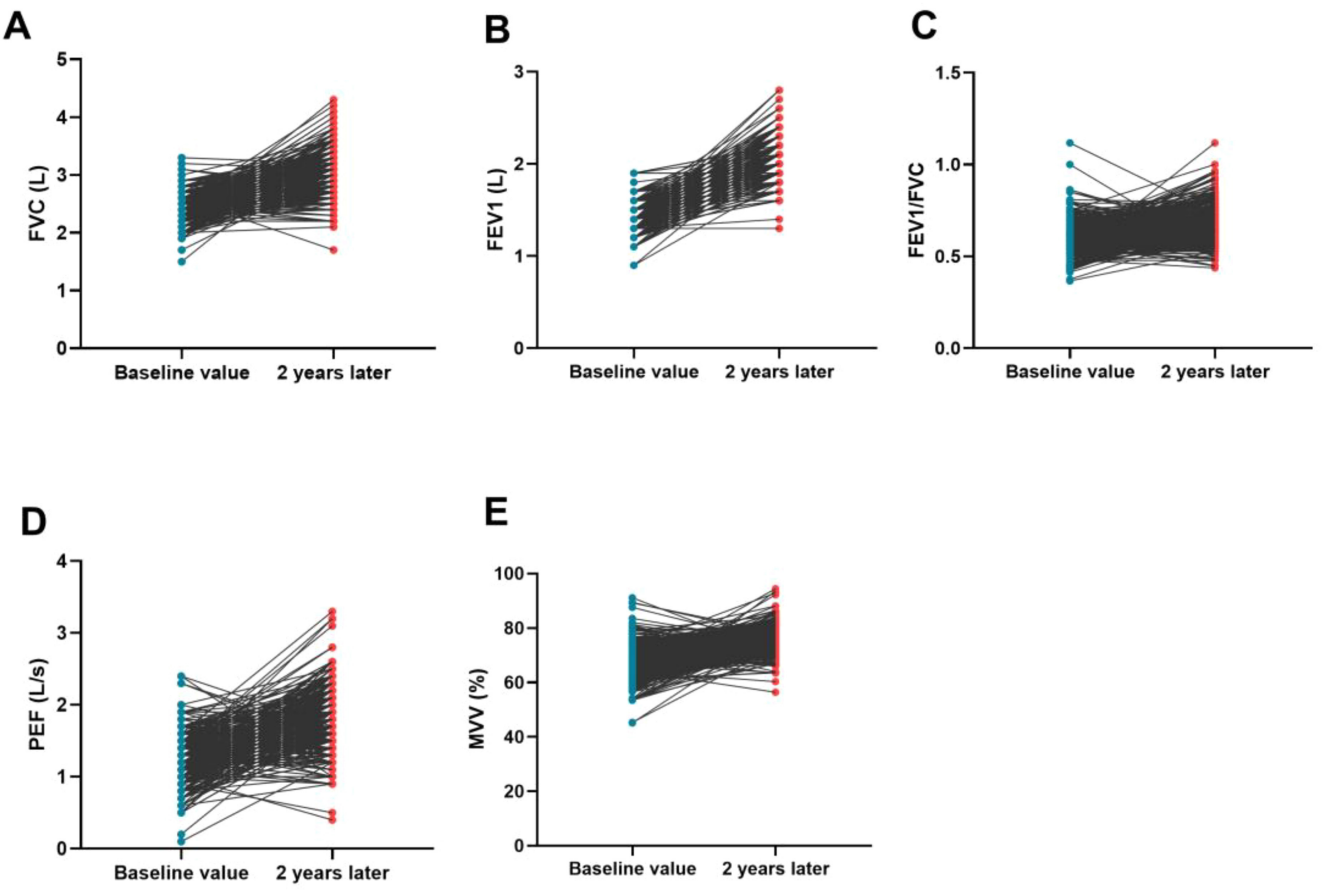
Changes in IoT-driven dynamic physiological indicators between baseline and 2-year follow-up in COPD patients. **(A)** FVC; **(B)** FEV1; **(C)** FEV1/FVC; **(D)** PEF; **(E)** MVV. IoT, Internet of Things; COPD, Chronic obstructive pulmonary disease; FVC, Forced vital capacity; FEV1, Forced expiratory volume in 1 s; PEF, Peak expiratory flow; MVV, Maximal voluntary ventilation.

Analysis of IoT-driven dynamic physiological monitoring metrics identified the five indicators exhibiting the most substantial changes over the 2-year period: FVC, FEV_1_, FEV_1_/FVC ratio, PEF, and MVV. Results from the 360 COPD patients revealed the following baseline values: median FVC: 2.40 L (IQR: 2.30, 2.60); median FEV_1_: 1.40 L (IQR: 1.30, 1.50); median FEV_1_/FVC ratio: 0.58 (IQR: 0.54, 0.65); median PEF: 1.30 L/s (IQR: 1.00, 1.50); MVV (mean ± SD): 67.60% ± 6.64%. At the 2-year follow-up, significant improvements were observed: median FVC: 3.10 L (IQR: 2.80, 3.30); median FEV_1_: 2.00 L (IQR: 1.90, 2.20); median FEV_1_/FVC ratio: 0.67 (IQR: 0.59, 0.74); median PEF: 1.80 L/s (IQR: 1.60, 2.10); MVV (mean ± SD): 76.13% ± 5.31%. Statistical analysis demonstrated highly significant differences between baseline and 2-year values for all five indicators (FVC: *Z* = −15.861; FEV_1_: *Z* = −16.404; FEV_1_/FVC: *Z* = −10.086; PEF: *Z* = −14.251; MVV: *Z* = −18.651; all *P* < 0.001). These results collectively indicate significant longitudinal changes in pulmonary function-related physiological metrics during follow-up (Figure 3 and Table 3).

**TABLE 3:** Changes in IoT-driven dynamic physiological indicators in COPD patients (baseline vs. 2-year follow-up) [M (P_25_, P_75_)].

Timepoint	FVC (L)	FEV_1_ (L)	FEV_1_/FVC	PEF (L/s)	MVV (%)
Baseline	2.40 (2.30, 2.60)	1.40 (1.30, 1.50)	0.58 (0.54, 0.65)	1.30 (1.00, 1.50)	67.60 ± 6.64
2-year	3.10 (2.80, 3.30)	2.00 (1.90, 2.20)	0.67 (0.59, 0.74)	1.80 (1.60, 2.10)	76.13 ± 5.31
Z	−15.861	−16.404	−10.086	−14.251	−18.651
*P*	<0.001	< 0.001	<0.001	< 0.001	<0.001

IoT, Internet of Things; COPD, Chronic obstructive pulmonary disease; FVC, Forced vital capacity; FEV_1_, Forced expiratory volume in 1 s; PEF, Peak expiratory flow; MVV, Maximal voluntary ventilation.

At the 2-year follow-up, 134 patients (37.22%) had a post-bronchodilator FEV_1_/FVC ratio ≥ 0.70 and therefore no longer fulfilled the fixed-ratio spirometric criterion for persistent airflow limitation according to GOLD. These patients had met the GOLD spirometric diagnostic criterion at baseline and were retained in the longitudinal trajectory analysis. Therefore, the observed follow-up spirometric values were interpreted cautiously as part of dynamic monitoring trends rather than as evidence of COPD reversal.

### 3.4 Univariate analysis of factors influencing QoL in COPD patients

The mean total QoL score at the 2-year follow-up was 81.45 ± 4.32 points. Based on this score, the 360 patients were stratified into a good QoL group (total score > 81 points, *n* = 184) and a poor QoL group (total score ≤ 81 points, *n* = 176). Univariate analysis of clinical characteristics revealed no statistically significant differences between the two groups regarding age, gender, educational attainment, marital status, occupation, place of residence, secondhand smoke exposure, family history of respiratory diseases, or baseline pulmonary function indicators (FVC, FEV_1_, FEV_1_/FVC ratio, PEF, and MVV; all *P* > 0.05). Conversely, statistically significant differences were observed between the good and poor QoL groups for smoking status, cumulative smoking load, occupational dust/harmful gas exposure, biomass fuel use, and rates of change in pulmonary function indicators including FVC, FEV_1_, and MVV (all *P* < 0.05), suggesting potential exploratory associations between these variables and QoL outcomes in COPD patients (Table 4).

**TABLE 4:** Univariate analysis of factors associated with QoL in COPD patients [$\bar{x}$ ± S, n, M (P_25_, P_75_)].

Group	Poor QoL group (*n* = 176)	Good QoL group (*n* = 184)	χ^2^/*t/z*	*P*
Age (years)	64.20 ± 8.63	63.22 ± 9.17	1.051	0.294
Gender			0.943	0.332
Male	126	140		
Female	50	44		
Educational attainment			0.245	0.620
Junior high school or below	131	122		
High school or above	55	62		
Marital status			0.031	0.859
Married	149	157		
Single/divorced/widowed	27	27		
Occupation			2.745	0.601
Service industry	15	23		
Other laborers	39	34		
Retired	85	88		
Professional/technical	21	26		
Unemployed	16	13		
Residence			1.274	0.259
Urban	86	79		
Rural	90	105		
Smoking history	141	92	35.724	<0.001
Cumulative smoking (pack-years)			34.465	<0.001
0∼30	90	148		
>30	86	36		
Secondhand smoke exposure			1.098	0.295
Yes	15	49		
No	20	43		
Occupational dust/harmful gas exposure	88	50	19.827	<0.001
Biomass fuel use	88	70	5.222	0.022
Family history of respiratory diseases	60	58	0.269	0.604
Personal history of respiratory diseases	64	46	5.474	0.019
FVC-baseline (L)	2.40 (2.30, 2.50)	2.40 (2.30, 2.60)	−0.952	0.341
FEV_1_-baseline (L)	1.40 (1.30, 1.50)	1.40 (1.30, 1.50)	−0.598	0.550
FEV_1_/FVC-baseline	0.60 (0.54, 0.65)	0.57 (0.52, 0.65)	−1.121	0.262
PEF-baseline (L/s)	1.20 (1.00, 1.50)	1.30 (1.10, 1.50)	−0.636	0.525
MVV-baseline (%)	68.01 ± 6.82	57.21 ± 6.46	1.143	0.254
FVC change rate (%)	21.29 (8.61, 34.78)	33.97 (21.28, 45.83)	−5.570	<0.001
FEV_1_ change rate (%)	40.00 (28.57, 50.00)	50.00 (33.33, 64.29)	−4.541	< 0.001
FEV_1_/FVC change rate (%)	17.33 (1.68, 33.62)	11.97 (−1.62, 31.67)	−1.081	0.280
PEF change rate (%)	42.86 (12.32, 100.00)	50.00 (21.07, 85.13)	−0.365	0.715
MVV change rate (%)	10.24 (2.97, 18.28)	15.44 (6.00, 23.34)	−2.666	0.008

QoL, Quality of life; COPD, Chronic obstructive pulmonary disease; FVC, Forced vital capacity; FEV_1_, Forced expiratory volume in 1 s; PEF, Peak expiratory flow; MVV, Maximal voluntary ventilation.

### 3.5 Multivariate analysis of factors influencing QoL in COPD patients

Multivariable logistic regression analysis was performed to identify independent predictors of QoL, with QoL (poor QoL = 0, good QoL = 1) serving as the dependent variable. The independent variables included smoking status, cumulative smoking load, occupational dust/harmful gas exposure, biomass fuel use, and personal history of respiratory diseases (variable coding scheme detailed in Table 5). No significant multicollinearity was identified among the included variables (all VIF values < 5). In addition, the Hosmer-Lemeshow goodness-of-fit test demonstrated satisfactory calibration of the final model (*P* > 0.05). The analysis revealed that smoking [odds ratio (*OR*) = 0.343, 95% confidence interval (*CI*): 0.193–0.610, *P* < 0.001), cumulative smoking load > 30 pack-years (*OR* = 0.404, 95% *CI*: 0.229–0.713, *P* = 0.002), occupational dust/harmful gas exposure (*OR* = 0.342, 95% *CI*: 0.210–0.556, *P* < 0.001), and biomass fuel use (*OR* = 0.439, 95% *CI*: 0.271–0.713, *P* = 0.001) were negatively associated with QoL in patients with COPD. Conversely, FVC change rate (*OR* = 1.041, 95% *CI*: 1.026–1.056, *P* < 0.001), FEV_1_ change rate (*OR* = 1.027, 95% *CI*: 1.014–1.040, *P* < 0.001), and MVV change rate (*OR* = 1.025, 95% *CI*: 1.007–1.044, *P* = 0.010) were positively associated with QoL. A personal history of respiratory diseases did not demonstrate a statistically significant independent association with QoL outcomes in this model (OR = 0.655, 95% CI: 0.392–1.095, *P* = 0.107) (Table 6).

**TABLE 5:** Variable coding scheme for multivariable logistic regression.

Variable	Coding
Dependent variable	Poor QoL = 0; Good QoL = 1
Independent variables	
Smoking status	Non-smoker = 0; Smoker = 1
Cumulative smoking	0–30 Pack-years = 0; > 30 pack-years = 1
Occupational dust/harmful gas exposure	No = 0; Yes = 1
Biomass fuel use	No = 0; Yes = 1
Personal history of respiratory diseases	No = 0; Yes = 1
FVC change rate	Continuous variable
FEV_1_ change rate	Continuous variable
MVV change rate	Continuous variable

QoL, Quality of life, FVC, Forced vital capacity; FEV_1_, Forced expiratory volume in 1 s; MVV, Maximal voluntary ventilation.

**TABLE 6:** Multivariable analysis of factors associated with QoL in COPD patients.

Independent variable	*B*	*SE*	Wald	*P*	*OR*	95% CI	
						Lower	Upper
Smoking status	−1.070	0.293	13.290	<0.001	0.343	0.193	0.610
Cumulative smoking	−0.907	0.290	9.785	0.002	0.404	0.229	0.713
Occupational dust/harmful gas exposure	−1.073	0.248	18.702	<0.001	0.342	0.210	0.556
Biomass fuel use	−0.823	0.247	11.088	0.001	0.439	0.271	0.713
Personal history of respiratory diseases	−0.423	0.262	2.604	0.107	0.655	0.392	1.095
FVC change rate	0.040	0.007	5.532	<0.001	1.041	1.026	1.056
FEV_1_ change rate	0.027	0.006	4.195	<0.001	1.027	1.014	1.040
MVV change rate	0.025	0.010	2.573	0.010	1.025	1.006	1.044

QoL, Quality of life; COPD, Chronic obstructive pulmonary disease; SE, Standard error; OR, Odds ratio; CI, Confidence interval; FVC, Forced vital capacity; FEV_1_, Forced expiratory volume in 1 s; MVV, Maximal voluntary ventilation.

As a sensitivity analysis, WHOQOL-BREF total score at the 2-year follow-up was additionally analyzed as a continuous outcome variable using exploratory linear regression. The results demonstrated generally consistent associations with the primary dichotomized analysis. Smoking status, cumulative smoking load, occupational dust/hazardous gas exposure, and biomass fuel use were independently associated with lower WHOQOL-BREF scores (all *P* < 0.05). In contrast, higher FVC and FEV_1_ change rates were independently associated with better QoL outcomes (both *P* < 0.001). However, respiratory disease history and MVV change rate were not independently associated with continuous WHOQOL-BREF scores (both *P* > 0.05). The overall regression model was statistically significant (Adjusted *R*^2^ = 0.190, *P* < 0.001), supporting the robustness of the observed associations between dynamic physiological indicators and QoL outcomes (Table 7).

**TABLE 7:** Sensitivity analysis table (continuous WHOQOL-BREF outcome).

Variables	β	SE	*t*	*P*-value	95% CI
Smoking status	−1.444	0.516	−2.798	0.005	−2.459 to −0.429
Smoking load (> 30 pack-years)	−1.206	0.512	−2.356	0.019	−2.212 to −0.199
Occupational dust/hazardous gas exposure	−1.349	0.423	−3.19	0.002	−2.181 to −0.517
Biomass fuel use	−1.38	0.418	−3.303	0.001	−2.201 to −0.558
History of respiratory disease	0.013	0.456	0.028	0.978	−0.883 to 0.909
FVC change rate	0.039	0.01	3.993	< 0.001	0.020–0.058
FEV_1_ change rate	0.041	0.009	4.367	< 0.001	0.022–0.059
MVV change rate	0.015	0.014	1.026	0.305	−0.014–0.043

QoL, Quality of life; COPD, Chronic obstructive pulmonary disease; SE, Standard error; OR, Odds ratio; CI, Confidence interval, FVC, Forced vital capacity; FEV_1_, Forced expiratory volume in 1 s; MVV, Maximal voluntary ventilation. Model statistics: *R*^2^ = 0.208, Adjusted *R*^2^ = 0.190, *F* = 11.50, *P* < 0.001.

### 3.6 Diagnostic value analysis of factors influencing QoL in COPD patients

**FIGURE 4:**
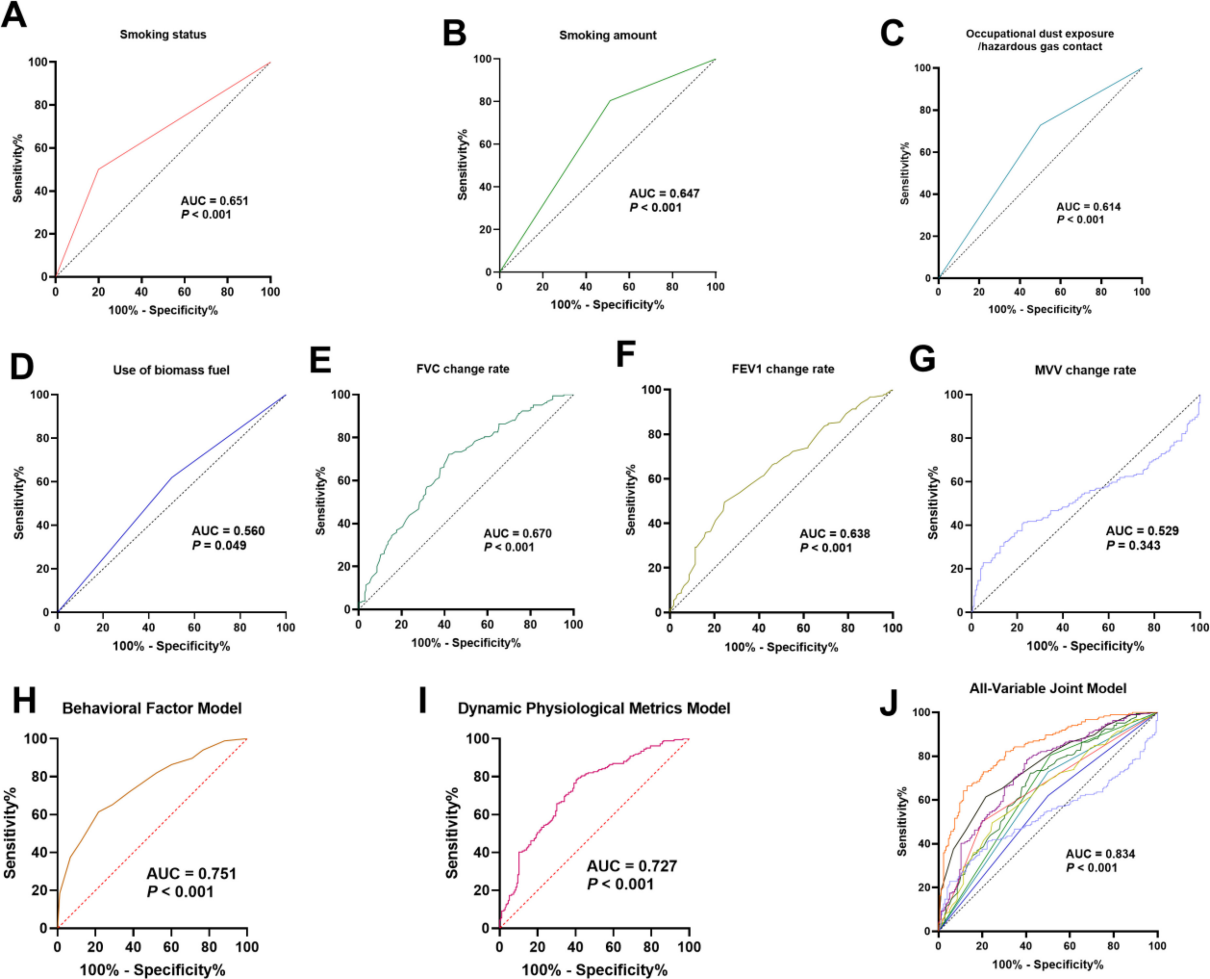
ROC analysis of factors associated with QoL in COPD patients. **(A)** Smoking status; **(B)** cumulative smoking load; **(C)** occupational dust/harmful gas exposure; **(D)** biomass fuel use; **(E)** FVC change rate; **(F)** FEV1 change rate; **(G)** MVV change rate; **(H)** behavioral factor model; **(I)** dynamic physiological indicator model; **(J)** combined model. ROC, Receiver operating characteristic; QoL, Quality of life; COPD, Chronic obstructive pulmonary disease; FVC, Forced vital capacity; FEV1, Forced expiratory volume in 1 s; MVV, Maximal voluntary ventilation.

Analysis of the diagnostic performance of individual factors revealed the following: Smoking status yielded an area under the curve (AUC) of 0.651 (95% *CI*: 0.594–0.707, *P* < 0.001), cumulative smoking load (> 30 pack-years) had an AUC of 0.647 (95% *CI*: 0.589–0.704, *P* < 0.001), and biomass fuel use showed an AUC of 0.560 (95% *CI*: 0.500–0.619, *P* = 0.049). Among the dynamic physiological indicators, FVC change rate and FEV_1_ change rate also demonstrated predictive value (both *P* < 0.05), with diagnostic performance comparable to that of the behavioral factors. Although the predictive performance of individual dynamic physiological indicators did not substantially surpass that of traditional behavioral factors, the combined analysis revealed significant complementarity between the two. Further ROC curve analysis was conducted using three models: a behavioral factor model (including smoking, cumulative smoking load, occupational exposure, and biomass fuel use), a dynamic physiological indicator model (including FVC change rate, FEV_1_ change rate, and MVV change rate), and a combined model incorporating all variables. The AUC for the behavioral factor model was 0.751 (95% *CI*: 0.701–0.800, *P* < 0.001). The AUC for the dynamic physiological indicator model was 0.727 (95% *CI*: 0.675–0.779, *P* < 0.001). The combined model yielded an AUC of 0.834 (95% *CI*: 0.793–0.875, *P* < 0.001), which was superior to either individual model. These findings indicate that dynamic lung function information obtained through IoT-based monitoring provides additional predictive value beyond traditional risk factors, and that combining multidimensional indicators significantly improves the ability to identify patients with COPD at risk for poor QoL (Figure 4).

## 4 Discussion

This study leveraged an IoT platform to conduct longitudinal, continuous physiological monitoring and remote management, focusing on QoL as a patient-centered outcome. Our analysis revealed an overall improvement in pulmonary function metrics, represented by FVC, FEV_1_, FEV_1_/FVC ratio, PEF, and MVV, during the follow-up period. Furthermore, the rates of change in FVC, FEV_1_, and MVV were retained in the multivariate model, suggesting that dynamic changes in pulmonary function not only reflect functional evolution during follow-up but are also closely associated with QoL outcomes. These findings suggest that continuous monitoring coupled with timely health education and medication guidance may be associated with improved airflow limitation and ventilatory reserve. However, QoL exhibited a divergent trajectory across its four domains: gains in physical and environmental dimensions contrasted with declines in psychological health and social relationships. This indicates that physiological and environmental improvements alone are insufficient to drive parallel gains in psychological and social wellbeing. Importantly, one of the most clinically meaningful findings of this study was that improvements in pulmonary function parameters did not translate into parallel improvements in overall QoL. Although IoT-enabled monitoring and management were associated with better physiological indicators and environmental domain scores, the psychological and social relationship domains, as well as the total QoL score, showed a declining trend over time. This discrepancy suggests that physiological improvement alone may be insufficient to fully improve patient-perceived wellbeing in COPD. QoL in COPD is inherently multidimensional and is influenced not only by airflow limitation and ventilatory function, but also by psychological burden, social isolation, behavioral adaptation, chronic disease perception, and digital health engagement. Therefore, COPD management strategies based solely on physiological optimization may overlook important patient-centered outcomes. Smoking, cumulative smoking load, occupational dust/harmful gas exposure, and biomass fuel use were significantly associated with poor QoL, while a combined predictive model demonstrated moderate discriminative power for identifying at-risk patients. Notably, baseline pulmonary function levels did not show consistent discriminative ability between the two groups, whereas dynamic rates of change had clearer predictive value. This suggests that differences in QoL among patients with COPD may not depend primarily on baseline pulmonary function levels but may be more closely related to the direction and magnitude of functional changes during follow-up. Collectively, these findings suggest the potential clinical relevance of an IoT-enabled physiological monitoring-behavioral intervention-contextual management pathway while underscoring that multidimensional QoL enhancement requires synergistic integration of physiological, psychological, and social support strategies.

The physiological value of IoT manifests primarily in its capacity to identify early deterioration signals (e.g., daily declines in PEF/FEV_1_, increased nocturnal variability) through continuous trajectory analysis, potentially facilitating earlier clinical responses or follow-up adjustments, such as health education, medication adjustments, and follow-up reassessment, days before acute exacerbations develop (19). By referencing individualized historical baselines and integrating symptom self-reports with objective activity data, IoT helps distinguish true clinical worsening from artifacts (e.g., measurement variability or suboptimal effort), thereby reducing unnecessary medical interventions (20). The statistical stability afforded by high-frequency sampling allows detection and reinforcement of subtle yet clinically meaningful improvements (21). It is noteworthy that, under real-world conditions, this study observed an overall improvement in pulmonary function indicators, with the dynamic physiological indicator model achieving an AUC of 0.728, suggesting that relying solely on dynamic pulmonary function information provides a certain level of QoL risk identification capability. This suggests that IoT-driven remote monitoring may be associated with favorable physiological trends during longitudinal follow-up without substantially increasing the burden of hospital visits. However, sustained patient adherence remains an important practical challenge in long-term IoT-based monitoring. Older patients with COPD may experience difficulties related to digital literacy, device fatigue, irregular usage behavior, or declining engagement over prolonged follow-up, potentially affecting data completeness and monitoring reliability. Therefore, future IoT management strategies should emphasize user-friendly device design, patient education, automated reminders, and long-term engagement mechanisms to improve adherence and data quality. In the present study, the rates of change in FVC, FEV_1_, and MVV were retained in the final model, whereas FEV_1_/FVC and PEF were not independently associated with QoL despite showing overall improvement during follow-up. This finding suggests that pulmonary function parameters reflecting ventilatory reserve and the magnitude of functional change may provide greater insight into QoL risk than isolated static measurements. Nevertheless, the magnitude of spirometric improvement observed over the 2-year follow-up warrants cautious interpretation. COPD is characterized by persistent airflow limitation, and the substantial increases in FEV_1_, FVC, and FEV_1_/FVC are unlikely to be explained solely by physiological recovery. Recent GOLD 2026 updates further emphasize (22) that COPD should be regarded as a heterogeneous clinical condition incorporating respiratory symptoms, exposure history, structural abnormalities, and functional impairment rather than relying solely on spirometric thresholds. Therefore, the fixed-ratio criterion should be interpreted cautiously within the broader clinical context, particularly in elderly community-based populations where age-related physiological decline in lung function may increase the risk of overdiagnosis. Several alternative explanations should therefore be considered. Repeated spirometric assessments may have improved patient familiarity and expiratory effort, particularly among elderly individuals undergoing portable spirometry testing. Continuous health education, inhaler technique guidance, smoking reduction, and enhanced disease awareness during follow-up may also have contributed to partial functional improvement. In addition, measurement variability inherent to portable spirometric devices may have influenced longitudinal changes. Although spirometric procedures were conducted using standardized devices and trained personnel in accordance with ATS/ERS recommendations, portable spirometry may still be associated with greater measurement uncertainty compared with laboratory-grade pulmonary function systems, which could have contributed to systematic variability in the observed rates of change. Therefore, the present study focused primarily on longitudinal rates of change and trajectory-based physiological trends rather than isolated absolute spirometric values, as dynamic changes may better reflect real-world functional evolution and patient-centered outcomes in IoT-based monitoring settings. Although patients with previously diagnosed asthma were excluded at enrollment, the possibility of undetected reversible airflow obstruction or asthma-COPD overlap in a subset of participants cannot be completely excluded.

Psychological and social QoL dimensions are influenced by multifactorial drivers. Enhanced disease awareness and vigilance toward IoT-generated alerts may exacerbate health anxiety in some patients (23). Older adults with chronic diseases often exhibit fragile digital literacy and social networks. Without embedded psychological support and peer-connection modules, IoT systems risk creating a “data-rich yet support-poor” mismatch (24). External factors during the study period (e.g., social restrictions during public health crises, familial caregiving strain) may further amplify negative perceptions. Thus, IoT solutions must evolve from a tool-centric to a patient-centric, team-empowered paradigm: beyond monitoring and alerts, integrating behavioral science-informed micro-interventions (e.g., cognitive behavioral therapy modules, relaxation training, sleep hygiene guidance), establishing rapid-access online psychological counseling and peer-support groups, and leveraging gamified goal-setting with positive feedback in patient apps to transform passive monitoring into active rehabilitation (25–27). In this study, although patients showed improvements in the physical and environmental domain scores, the psychological and social relationship domain scores, as well as the total QoL score, declined. These findings indicate that physiological improvement alone may be insufficient to achieve comprehensive QoL benefits. In the present study, although physical and environmental domain scores showed improvement over time, psychological and social relationship domain scores, as well as overall QoL, demonstrated a declining trend. A primary explanation for this divergence may be the natural progression of COPD itself, as chronic symptom burden, progressive functional limitation, reduced social participation, and long-term disease-related stress are known to adversely affect psychological and social wellbeing over time. In addition, several other factors may also have contributed to these findings, including increased awareness of health status during continuous monitoring, persistent concerns regarding disease progression, reduced digital adaptability among elderly individuals, and potential limitations in social support resources. However, these potential mechanisms were not directly assessed in the present study and should therefore be interpreted cautiously. These findings suggest that long-term COPD management should not only focus on physiological stabilization but also address emotional wellbeing, social connectedness, and patient self-efficacy.

Smoking, high cumulative pack-years, occupational dust/harmful gas exposure, and biomass fuel use, all modifiable risk factors (28, 29), demonstrated significant associations with poor QoL. IoT platforms can operationalize “smoking cessation-exposure control-health education” pathways through synchronized interventions: linking Bluetooth-enabled carbon monoxide monitors or nicotine replacement therapy adherence tracking to lung function trends for personalized feedback (30); deploying geofencing and environmental sensors to identify high-exposure scenarios and trigger real-time avoidance reminders; and providing step-by-step ventilation/stove-modification guidance for biomass fuel users, with pre/post-implementation tracking of indoor air quality and symptoms (31, 32). Critically, IoT closes the “lifestyle-exposure-physiological change-QoL feedback” loop, enabling patients to visualize immediate and intermediate benefits of behavioral changes, thereby strengthening intrinsic motivation for sustained action (33). The results further showed that the behavioral factor model achieved an AUC of 0.751, higher than that of the dynamic physiological indicator model (0.727), whereas the combined model incorporating all variables achieved an AUC of 0.834, which was superior to either model alone. These findings suggest that behavioral, environmental, and physiological factors provide complementary information for QoL risk assessment. In other words, relying solely on smoking and exposure information or solely on dynamic pulmonary function changes is insufficient to fully explain patient QoL risk, whereas combining the two enables more comprehensive identification of individuals at high risk for poor QoL, which better reflects the true nature of COPD as a disease shaped by the interaction of behavior, environment, and physiology. These findings further support the concept that QoL impairment in COPD is driven by the interaction of physiological, behavioral, environmental, and potentially psychosocial factors rather than any single domain alone. Therefore, multifactorial and patient-centered risk assessment strategies may provide greater clinical value than approaches relying solely on isolated physiological indicators.

The moderate discriminative accuracy of the multifactor model supports the feasibility of integrating lifestyle, environmental exposure, and physiological time-series data for risk stratification. Clinical implementation can be optimized by: (1) augmenting static metrics (e.g., FVC/FEV_1_) with dynamic temporal features (3–7-day moving averages, coefficients of variation, nocturnal minima, morning-evening differences) and symptom-event density/duration (cough/sputum/wheezing); (2) replacing fixed thresholds with intra-individually standardized Z-scores or percentile shifts to enhance robustness in cross-patient comparisons; and (3) prioritizing interpretable models [logistic regression/gradient boosting with SHapley Additive exPlanations (SHAP) values] to clarify feature contributions for clinician-patient communication. While advanced techniques (mixed-effects or state-space/change-point detection models) may improve early detection as datasets expand, interpretability and actionability must remain paramount to avoid “black-box” limitations. Thus, the additional findings from this study not only strengthen the completeness of the results but also highlight, from a methodological perspective, the importance of extracting and utilizing dynamic trajectory features in COPD remote management research over single baseline measurements.

### 4.1 Strengths and limitations

Key strengths in our study include: (1) continuous monitoring capturing real-world patient experiences; (2) joint assessment of physiological and QoL outcomes, aligning with patient-centered care principles; and (3) focus on modifiable factors (smoking/exposure) to inform actionable management. The analysis of dynamic rates of change and the comparison of predictive models allow this study to move beyond simple identification of associated factors and demonstrate the potential for risk prediction and stratified management. However, several important limitations should be acknowledged. First, this was a single-arm observational cohort study without a concurrent control group, which limits causal inference regarding the independent effects of IoT-based monitoring on pulmonary function and QoL outcomes. Accordingly, the observed longitudinal changes should be interpreted as associative rather than causal and may also have been influenced by factors such as repeated spirometry familiarity, increased clinical attention, behavioral modification, or natural disease progression. Second, potential information bias related to device non-adherence, irregular usage behavior, missing monitoring data, and the retrospective complete-case analytical framework may have introduced selection bias. Third, the dichotomization of QoL based on the sample mean WHOQOL-BREF score was exploratory in nature and may have reduced statistical sensitivity and external generalizability. Although supplementary sensitivity analyses using continuous WHOQOL-BREF outcomes demonstrated generally consistent findings, the absence of externally validated QoL cut-points and the lack of formal correction for multiple comparisons may still have increased the possibility of false-positive findings. Fourth, several important confounders, including acute exacerbation frequency, GOLD severity grade, chronic comorbidities, medication adherence, healthcare utilization, and medical costs, were not systematically incorporated because of data availability limitations, potentially contributing to residual confounding and intergroup heterogeneity. Fifth, psychological and social determinants such as emotional support, social isolation, coping style, and digital literacy were insufficiently captured, which may partially explain the divergence between physiological improvement and declines in psychological and social QoL domains. Finally, this study was conducted in a single geographic region, potentially limiting generalizability, and emerging GOLD 2026 concepts such as Pre-COPD and PRISm were not specifically evaluated. Although the combined predictive model demonstrated good discriminative performance, external validation was not performed. Therefore, future multicenter, controlled, and externally validated prospective studies incorporating comprehensive psychosocial assessment, health economic analyses, and long-term clinical endpoints are still required.

## 5 Conclusion

This study suggests that IoT-based monitoring may provide a potential framework for continuous monitoring, timely feedback, and stratified management in COPD care. The observed associations between dynamic pulmonary function trajectories and QoL outcomes suggest the potential clinical relevance of IoT-based monitoring in COPD management. Notably, dynamic pulmonary function indicators, particularly rates of change in FVC, FEV_1_, and MVV, are independently associated with QoL outcomes, and their combination with behavioral and exposure factors further improves the predictive accuracy for poor QoL, highlighting the value of dynamic monitoring data in patient risk stratification. However, multidimensional QoL improvement is not an automatic byproduct of physiological gains. It necessitates systematic integration of psychological support, social connectivity, and targeted interventions for smoking and environmental exposures within IoT architectures. These findings further emphasize that patient-centered COPD management should extend beyond physiological optimization and incorporate comprehensive psychosocial and behavioral support strategies. With standardized implementation and augmented health economic evidence, IoT-based monitoring may represent a potentially valuable supportive framework for community-based COPD management. However, further multicenter controlled studies are required to determine its long-term clinical effectiveness, scalability, and health-economic value.

## Data Availability

The original contributions presented in the study are included in the article/supplementary material, further inquiries can be directed to the corresponding authors.
